# Beta-nodavirus B2 protein induces hydrogen peroxide production, leading to Drp1-recruited mitochondrial fragmentation and cell death via mitochondrial targeting

**DOI:** 10.1007/s10495-014-1016-x

**Published:** 2014-07-10

**Authors:** Yu C. Su, Hsuan W. Chiu, Jo C. Hung, Jiann R. Hong

**Affiliations:** Laboratory of Molecular Virology and Biotechnology, Institute of Biotechnology, National Cheng Kung University, Tainan, 701 Taiwan, ROC

**Keywords:** Anti-oxidants, Cell death, Hydrogen peroxide production, Mitochondrial fragmentation, Nervous necrosis virus, Oxidative stress

## Abstract

Because the role of the viral B2 protein in the pathogenesis of nervous necrosis virus infection remains unknown, the aim of the present study was to determine the effects of B2 protein on hydrogen peroxide (H_2_O_2_)-mediated cell death via mitochondrial targeting. Using a B2 deletion mutant, the B2 mitochondrial targeting signal sequence (^41^RTFVISAHAA^50^) correlated with mitochondrial free radical production and cell death in fish cells, embryonic zebrafish, and human cancer cells. After treatment of grouper fin cells (GF-1) overexpressing B2 protein with the anti-oxidant drug, *N*-acetylcysteine (NAC), and overexpression of the antioxidant enzymes, zfCu/Zn superoxide dismutase (SOD) and zfCatalase, decreased H_2_O_2_ production and cell death were observed. To investigate the correlation between B2 cytotoxicity and H_2_O_2_ production in vivo, B2 was injected into zebrafish embryos. Cell damage, as assessed by the acridine orange assay, gradually increased over 24 h post-fertilization, and was accompanied by marked increases in H_2_O_2_ production and embryonic death. Increased oxidative stress, as evidenced by the up-regulation of Mn SOD, catalase, and Nrf2, was also observed during this period. Finally, B2-induced dynamin-related protein 1 (Drp1)-mediated mitochondrial fragmentation and cell death could be reversed by NAC and inhibitors of Drp1 and Mdivi in GF-1 cells. Taken together, betanodavirus B2 induces H_2_O_2_ production via targeting the mitochondria, where it inhibits complex II function. H_2_O_2_ activates Drp1, resulting in its association with the mitochondria, mitochondrial fission and cell death in vitro and in vivo.

## Introduction

Betanodavirus infection causes viral nervous necrosis (VNN) in fish, which is an infectious neuropathological condition characterized by necrosis of the central nervous system, including the brain and retina [1]. Clinical signs include abnormal swimming behavior and darkening of the fish [2]. VNN can induce massive death of the larval and juvenile populations of several marine teleost species [3]. However, little is known about the molecular pathogenesis of VNN.

The nodavirus genome comprises two single-stranded molecules of positive polarity, RNA1 and RNA2 that are approximately 3.1 and 1.4 kb in length, respectively [4]. RNA1 encodes a nonstructural protein of approximately 110-kDa, designated RNA-dependent RNA polymerase or protein A that is vital for replication of the viral genome [4]. RNA2 encodes a 42-kDa capsid protein [4, 5] that may induce post-apoptotic necrotic cell death through a pathway mediated by cytochrome *c* release [6]. During RNA replication, betanodaviruses synthesize a sub-genomic RNA3 from the 3′ terminus of RNA1 that encodes two non-structural proteins, B1 and B2 [1, 7, 8]. In red-spotted grouper nervous necrosis virus (RGNNV), B1 has anti-necrosis functions [9]; B2 acts as a host siRNA silencing suppressor in alphanodavirus [10–12] and betanodavirus [7].

Oxidative stress has been implicated in the pathogenesis of neurodegenerative diseases, such as Alzheimer’s and Parkinson’s diseases [13, 14]. Oxidative stress occurs in cells when production of reactive oxygen species (ROS) exceeds the cell’s endogenous antioxidant defenses [15]. The major cellular defenses against ROS include superoxide dismutases (SODs) and catalase [16, 17]. SODs catalyze the dismutation of superoxide (O_2_^−^) to hydrogen peroxide (H_2_O_2_) and molecular oxygen (O_2_) and are located in the cytoplasm (Cu/Zn SOD) and mitochondria (Mn SOD) [18, 19].

The induction of apoptosis and post-apoptotic necrotic cell death mediated by mitochondrial membrane potential loss and cytochrome c release by the RGNNV TN1 strain in fish cells was first identified by Chen et al. [20]. Necrosis was blocked by the mitochondrial membrane permeability transition pore inhibitor, bongkrekic acid (BKA) [20], the anti-apoptotic Bcl-2 family member protein, zfBcl-xL [9], and the protein synthesis inhibitor, cycloheximide [21], suggesting that necrosis requires the synthesis of new protein. In addition, b2 protein can induce Bax-mediated cell death [12] and cause ATP depletion via blocking complex II function [22]. B2-induced Bax-mediated necrotic cell death can be blocked by overexpression of zfBcl-xL [8, 12]. Furthermore, we recently found that the RGNNV TN1 strain can induce ROS production, triggering the oxidative stress response [23]. However, the reason for this observation remains unknown. Therefore, this study aimed to elucidate the role of the B2 protein in the pathogenesis of betanodavirus infection in fish. In particular, we investigated the effects of B2 protein on oxidative stress-mediated cell death via mitochondrial targeting in vitro and in vivo.

## Materials and methods

### Cells

The grouper cell line, GF-1, was obtained from Dr. Chi (Institute of Zoology and for the Development of Life Science, Taiwan, ROC). Cells were maintained at 28 °C in Leibovitz’s L-15 medium (GibcoBRL, Gaithersburg, MD, USA) supplemented with 5 % fetal bovine serum (GeneDireX, San Diego, CA, USA) and 25 μg/mL gentamycin (GibcoBRL). Human embryonic kidney cell line (293T cells), epithelial cervical cancer cells (HeLa cells), breast adenocarcinoma cells (MCF-7 cells), lung adenocarcinoma cells (A549 cells and H1299 cells) were grown at 37 °C in low glucose Dulbecco’s modified Eagle’s medium (DMEM, GibcoBRL) supplemented with 10 % fetal bovine serum and 5 % CO_2_.

### Plasmid construction and cell transfection

The B2 coding sequence and mitochondrial targeting signal deletion fragments were cloned into the p3XFlag-myc-CMV-26 (Sigma, St. Louis, MO, USA) or pEYFP-C1 (Clontech Laboratories, Mountain View, CA, USA) vectors, and sequenced to verify the reading frame as previously described [22] (Table 1).

**Table 1:** The sequence primers used in this study

Name	Primers^a^
pEYFP-B2(1-70)	
Forward	GAAGATCTATGGAACAAATCCAACAA (*Bgl*II)
Reverse	GGAATTCCTAGTCCGTCTCCATCGG (*Eco*RI)
pEYFP-B2(Δ)	
Forward 1	GAAGATCTATGGAACAAATCCAACAA (*Bgl*II)
Reverse 1	GGAATTCCTACATCTCGTTTACCTGGAT (*Eco*RI)
Forward 2	GGAATTCGCTCGCCGCCTGCACGTC (*Eco*RI)
Reverse 2	CGGGATCCCTAGTCCGTCTCCATCGG (*Bam*HI)
p3XFLAG-B2	
Forward	GAAGATCTGATGGAACAAATCCAACAA (*Bgl*II)
Reverse	CGGGATCCCTAGTCCGTCTCCATCGG (*Bam*HI)
p3XFLAG-B2(Δ)	
Forward	GAAGATCTGATGGAACAAATCCAACAA (*Bgl*II)
Reverse	CGGGATCCCTAGTCCGTCTCCATCGG (*Bam*HI)
zfCatalase	
Forward	TAAAGGAGCAGGAGCGTTTGGCTA
Reverse	TTCACTGCGAAACCACGAGGATCT
zfMn SOD	
Forward	CCGGACTATGTTAAGGCCATCT
Reverse	ACACTCGGTTGCTCTCTTTTCTCT
zfNrf2	
Forward	GAGCGGGAGAAATCACACAGAATG
Reverse	CAGGAGCTGCATGCACTCATCG
zfGAPDH	
Forward	CCAGGTTGTGTCCACTGACTT
Reverse	CATGTAATTTCCTTCCAGGCA
zfCu/Zn SOD	
Forward	GTCGTCTGGCTTGTGGAGTG
Reverse	TGTCAGCGGGCTAGTGCTT

^a^The enzymatic cutting sites on nucleotides of primers are underlined

For cell transfection, 3 × 10^5^ GF-1 cells were seeded in 60-mm diameter culture dishes. On the following day, 2 μg of recombinant plasmid was mixed with Lipofectamine 2000 (Invitrogen, Carlsbad, CA, USA), and the transfection procedure was carried out according to the manufacturer’s instructions.

### Western blot analysis

GF-1 cells were seeded in 60-mm diameter culture dishes with 3 mL medium (10^5^ cells/mL). At the end of each incubation period, the culture medium was aspirated, and the cells were washed with PBS and then lysed in 0.3 mL of lysis buffer (10 mM Tris, pH 6.8, 20 % glycerol, 10 mM sodium dodecyl sulfate (SDS) [24], 2 % ß-mercaptoethanol). An aliquot of each lysate with 30 μg protein per sample was separated by electrophoresis on an SDS polyacrylamide gel to resolve the proteins. The gels were immunoblotted with the following antibodies: (1) anti-Flag primary monoclonal antibodies (1:8,000 dilution; Sigma) followed by peroxidase-labeled goat anti-mouse secondary antibodies (1:15,000 dilution; Amersham Biosciences, Piscataway, NJ, USA) or (2) human anti-Dynamin 1-like protein (Drp1) or voltage-dependent anion channels (VDAC) primary polyclonal antibodies (1:1,500 dilution; Novus Biologicals, Littleton, CO, USA) followed by a peroxidase-labeled goat anti-rabbit secondary antibodies (1:7,500 dilution; Amersham Biosciences, Piscataway, NJ, USA). Chemiluminescence indicative of antibody binding was captured on Kodak XAR- 5 films (Eastman Kodak, New York, USA) as previously described [25].

### ROS production in intact cells

ROS levels were determined in living cells using the Image-iT LIVE Green Reactive Oxygen Species Detection Kit (Molecular Probes, Eugene, OR, USA), which uses carboxy-H_2_DCFDA (5-[and-6-]-carboxy-2′,7′-dichlorodihydrofluorescein diacetate) staining, a reliable fluorogenic marker of ROS formation in live cells. Cells cultured in 60-mm diameter dishes were transfected with Flag, Flag-B2, or Flag-B2Δ for 48 and 72 h. After the cells were gently washed once with PBS, 500 μL of a working solution of 25 μM carboxy-H_2_DCFDA (Life Technologies, Carlsbad, CA, USA) in PBS was added to the cells and incubated in the dark for 30 min. The samples were examined immediately under a fluorescence microscope with 100 W halogen for 0.5 s using the following band-pass filters: 488-nm excitation and 515-nm long-pass filter for detection of the fluorescein. The percentage of 200 cells at each time point was determined in triplicate, with each point representing the mean of three independent experiments.

To assess the total fluorescence, cells cultured on 6-well plates for 48 and 72 h post-transfection were incubated in the dark for 30 min with 350 μL of 25 μM carboxy-H_2_DCFDA in PBS. Total fluorescence per sample was determined with a fluorescence microplate reader using the following band-pass filters: 488-nm excitation and 515-nm long-pass filter for detection of the fluorescein as previously described [28].

### Hydrogen peroxide assay

Cellular H_2_O_2_ levels were determined using the Amplex Red Hydrogen Peroxide/Peroxidase Assay Kit (Molecular Probes, Life Technologies; Carlsbad, CA, USA). GF-1 cells or Cu/Zn SOD and CAT-expressing GF-1 cells described previously [23] (10^5^/mL) were cultured to monolayer confluence in 60-mm diameter culture dishes for 20 h, rinsed twice with PBS, and transfected with one of three plasmids (pFlag, pFlag-B2, or pFlag-B2Δ) and then incubated at 28 °C for 0, 24, or 48 h. After the medium was removed, the cells were washed with PBS, lysed in 0.1 mL of lysis buffer (50 mM Tris HCl, pH 7.4, 150 mM NaCl, 1 m M EDTA, 1 % Triton X-100, 0.5 mM PMSF) with shaking at 4 °C for 30 min. After centrifugation at 13,000 rpm for 2 min at 4 °C to pellet the insoluble materials, 50 μL of supernatant was mixed with 100 μM Amplex Red and 0.2 U/mL horseradish peroxidase and incubated at room temperature for 30 min in the dark. Fluorescence was measured in a microplate reader with excitation at 570 nm and fluorescence emission detection at 585 nm. Background fluorescence of the control (no H_2_O_2_) was subtracted from each reading.

### Propidium iodide (PI) staining

Cells (10^5^/mL) were cultured to monolayer confluence in 60-mm culture dishes for 20 h, rinsed twice with PBS, then transfected with either pFlag or pFlag-B2 for 2 h. Cells were then treated with either 1 mM NAC or 5 µM Mdivi (both Sigma) at 28 °C for 48 and 72 h. After the medium was removed, the monolayers were washed with PBS, incubated with 100 μL of PI (Boehringer-Mannheim Mannheim, Germany) in HEPES buffer for 10–15 min and evaluated using fluorescence microscopy as previously described [26].

### Selection of zebrafish Cu/Zn SOD and catalase-producing GF-1 cells

Zebrafish Cu/Zn SOD and catalase (zfCu/Zn SOD and zfCatalase, respectively) were cloned and inserted into the pBudCE4.1 expression vector (designated pBudCE4.1-zfCu/Zn SOD or pBudCE4.1-zfCatalase, respectively) by Dr. Ken [23]. pBudCE4.1- (negative control) and pBudCE4.1-zfCatalase-expressing cells were obtained by transfection of GF-1 cells and selection with Zeocin (500 μg/mL). Transcription of the inserted sequences was driven by the immediate-early promoter of human cytomegalovirus in these vectors. Selection time (2–2.5 months) varied based on cell-line-dependent properties.

### Flow cytometric analysis for cell viability

Viability analysis was conducting on the following cells: stained Flag and Flag-B2-transfected 293T cells, and a variety of cancer cell lines, including HeLa, MCF-7, A549, and H1299 cells (a p53−/− cell line). Analyses were performed using a FACS Vantage cell sorter (Becton–Dickinson, San Jose, CA, USA) to evaluate PI red fluorescence using a 650-nm long-pass bandpass filter. Altered cells have a higher PI fluorescence intensity (PI^+^) as compared to intact cells (PI^−^). At least 10,000 cells in the gated region were analyzed on the basis of light scatter properties. Fluorescence data were displayed on one or two major scales as previously described [22].

### Preparation of mitochondria from B2-transfected cells

GF-1 cells were seeded in 60-mm diameter culture dishes with 3 mL of medium (10^5^ cells/mL) for 20 h. GF1 cells were treated with 1 mM NAC and 5 µM Mdivi or were transfected with Flag, Flag-B2Δ and Flag-B2 for 48 h. At each change of the culture medium, 1 mL medium was removed. Mitochondria were isolated by modification of a previously described protocol [6]. Briefly, GF-1 cells (2 × 10^6^) were washed with PBS and homogenized in 0.3 mL of mitochondria isolation buffer (0.35 M mannitol, 10 mM HEPES, pH 7.2, 0.1 % bovine serum albumin) using a glass homogenizer. Unbroken cells and nuclei were pelleted by centrifugation (600×*g* for 5 min at 4 °C). The mitochondrial pellet was isolated by centrifugation (10,000×*g* for 10 min at 4 °C); the supernatant was collected and mixed with 25 µL of 10 × SDS sample buffer. Samples (50 µL) were boiled and subjected to Western blot analysis as previously described [25, 27].

### Maintenance of fish embryos in culture

Techniques for the care and breeding of zebrafish have been previously described in detail [28]. Embryos were collected from natural mating and maintained in embryonic medium (15 mM NaCl, 0.5 mM KCl, 1 mM CaCl_2_, 1 mM MgSO_4_, 0.05 mM Na_2_HPO_4_, 0.7 mM NaHCO_3_) at 28.5 °C. Embryos were staged according to standard morphological criteria [28].

### Microinjection of EYFP and EYFP-B2

To induce expression of the B2 protein in zebrafish embryos, 2 μL of a 10 ng/μL pEYFP-C1/pEYFP-B2 solution (linearized with EcoRI) was injected into each one-cell-stage embryo using a gas-driven microinjector (Medical System Corporation, Greenvale, NY, USA) as previously described [28].

### MitoTracker

To track changes in mitochondrial morphology, cells were transfected with pEYFP and pEYFP-B2 using Lipofectamine-Plus (Life Technologies) according to the manufacturer’s instructions and treated with 1 mM NAC and 10 μM Mdivi. Cells were then stained with MitoTracker Red CM-H_2_XRos (Invitrogen). Live cells were labeled with the mitochondrion-specific dye in accordance with the manufacturer’s instructions, after which cells were analyzed by fluorescence microscopy using 488 nm excitation and a 515 nm long-pass filter for green fluorescence and using 510 nm excitation and a 590 nm long-pass filter for red fluorescence previously described [21, 22].

### Immunostaining of Drp1 distribution in intact cells

GF-1 cells were seeded in 6-well plates with 2.5 mL of medium (10^5^ cells/mL) for 20 h and then transfected with EYFP, EYFP-B2 and EYFP-B2Δ for 48 h. The cells were washed with cold PBS, fixed in 4 % formaldehyde for 30 min at room temperature, washed with PBS twice and then permeabilized with PBST buffer (0.1 % Triton X-100 in PBS) for 15 min at room temperature. After the cells were washed with PBS twice, they were blocked with 1 % BSA for 60 min at RT, and then incubated overnight at 4 °C with antibodies against Drp1 (1:50, Aviva Systems Biology, San Diego, CA, USA) overnight. After cells were washed with PBST buffer twice, they were incubated for 60 min with Alexa Fluor^®^ 405 Goat Anti-Rabbit IgG (H+L) (1:500, Invitrogen) in 1 % BSA and washed with PBST twice. Immunofluorescence was examined using an Olympus IX70 fluorescence microscope (Tokyo, Japan) at 488-nm excitation and 515-nm long-pass filter for detection EYFP-B2 protein (with green fluorescence); 330-nm excitation and 420-nm long-pass filter was used for detection of the blue florescence (with Drp1) in the mitochondria.

### Quantification of cell viability

GF-1 cells expressing pBudCE4.1 (control), zfMn SOD-2, and zfCatalase-3 (all 10^5^ cells/mL) were cultured in 60-mm diameter culture dishes at 28 °C. After 0, 24, 48, or 72 h, cells were washed with PBS and treated with 0.5 mL of 0.1 % trypsin–EDTA (Gibco, Grand Island, NY, USA) for 1–2 min. Cell viability was determined in triplicate using the trypan blue dye exclusion assay as previously described [29]. Each data point (10,000 cells) represents the mean viability of three independent experiments ± SEM.

### Statistical analysis

The proportion of ROS production and PI-fluorescein-positive cells was determined in each sample by counting 200 cells. Each result is expressed as the mean ± SEM. Data were analyzed using either paired or unpaired Student’s *t* tests, as appropriate. A *P* value < 0.05 was considered a statistically significant difference between group mean values.

## Results

### Mitochondrial targeting of B2 protein is required for induction of free-radical species –H_2_O_2_ production

**Fig. 1:**
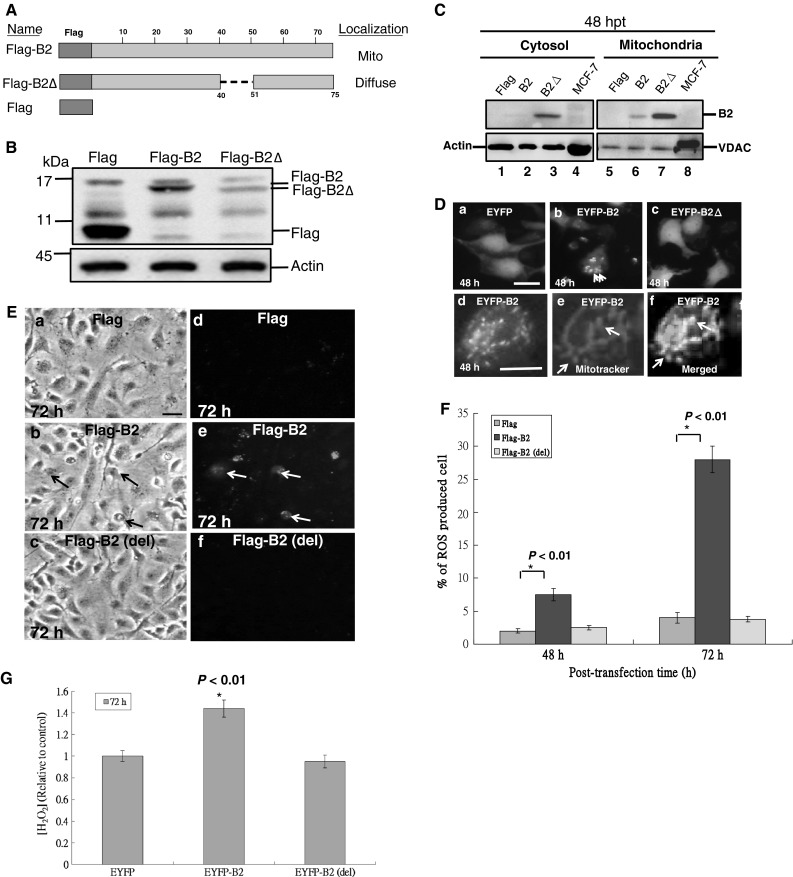
RGNNV B2 protein is targeted to the mitochondria and induces H_2_O_2_ production in fish cells. **A** Schematic representation of various full-length and mutant RGNNV B2 protein constructs used to identify the mitochondrial targeting sequence. **B** Immunoblot using monoclonal antibodies against the Flag tag shows the expression of various forms of Flag-B2. The internal control, actin, was shown at 48 h post-transfection. **C** Immunoblot using monoclonal antibodies against the Flag tag shows the protein distribution in either mitochondrial or cytosolic extracts at 48 h post-transfection. The internal controls included actin for the cytosolic fraction and VDAC for mitochondria membrane fraction. **D** GF-1 cells were transfected with EYFP, EYFP-B2 and EYFP-B2Δ, and their protein distribution was determined at 48 h post-transfection. EYFP-B2 protein was localized to mitochondria (**D**(b); indicated by *arrows*). GF-1 cells, transfected with EYFP-B2 (**D**(d); *green fluorescence*), were also stained with mitotracker at 48 h post-transfection (**D**(e); *red staining*); the merged image from **D**(d) and **D**(e) showed mitochondrial fragmentation indicated by *arrows* (**D**(f)). *Scale bar* 10 μm. **E** ROS production (*green* indicated by *arrows*) at 72 h post-transfection by cells transfected with RGNNV B2 (b and e). The negative control was Flag alone (a and d) and Flag-B2 (del^41^RTFVISAHAA^50^) (c and f). *Scale bar* 10 μm. **F** The percentage of ROS-producing cells at 48 and 72 h post-transfection. Error bars represent the SEM of three independent experiments. All data were analyzed using either a paired or unpaired Student’s *t* test, as appropriate. *P < 0.01. **G** Concentration of H_2_O_2_ in medium from EYFP-, EYFP-B2-, and EYFP-B2Δ-transfected cells at 72 h post-transfection. H_2_O_2_ concentration at each time point was determined in triplicate. All data were analyzed using either a paired or unpaired Student’s *t* test, as appropriate. *P < 0.01

The betanodavirus B2 protein has a specific signal peptide that targets it to mitochondria [22]. To determine whether mitochondrial targeting of the B2 protein is required for B2-mediated cell death, we constructed a *B2* gene with an N-terminal deletion that eliminates the signal sequence (del_40*-*51_; termed Flag-B2Δ) and fused it to a Flag tag (Fig. 1A). Recombinant plasmids were transfected into GF-1 cells and detected in whole cell lysates (Fig. 1B) as well as cytosolic or mitochondrial fractions (Fig. 1C) by Western blot analysis. Most B2 was localized to mitochondria (Fig. 1C, lane 6) as compared to the cytosol (Fig. 1C, lane 2). However, the B2Δ protein was localized to both the cytosol and mitochondria (Fig. 1C, lanes 3 and 7, respectively), suggesting that it may be anchored to the outer mitochondrial membrane through a yet unknown domain or structure. Separate immunofluorescence analysis confirmed that EYFP-B2 was primarily targeted to the mitochondria (Fig. 1D(b); indicated by arrows) as compared with EYFP alone (Fig. 1D(a)); EYFP-B2Δ was distributed throughout the cells (Fig. 1D(c)). EYFP-B2 targeting to mitochondria was confirmed using the mitochondria-specific dye, Mitotracker red (Fig. 1D(d–f); indicated by arrows).

In a ROS production assay, Flag-B2 expression induced ROS production as indicated by a punctate pattern and lethal morphological changes in cells producing ROS at 72 h post-transfection (Fig. 1E(b, e); indicated by arrows). However, cells expressing the Flag-B2Δ protein with the N-terminal deletion also showed a mild punctate pattern and the same morphology (Fig. 1E(c, f)) compared to those expressing Flag (Fig. 1E(a, d)). As shown in Fig. 1F, expression of the full-length B2 protein induced a 4.5 and 24 % increase in ROS production at 48 and 72 h post-transfection, respectively, when compared with vector alone (2 and 3.9 % at 48 and 72 h post-transfection, respectively) and the B2Δ mutant (3 and 3.8 % at 48 and 72 h post-transfection, respectively). Furthermore, as shown in Fig. 1G, expression of the full-length B2 protein induced H_2_O_2_ production by up to 1.48-fold as compared to either vector alone (baseline, 1-fold) or the B2Δ mutant (0.95-fold) at 72 h post-transfection. Taken together, these data indicate that residues 41–50 of the B2 protein target the protein to the mitochondria, where it plays a role in H_2_O_2_ production.

### B2 can trigger H_2_O_2_-mediated cancer cell death

**Fig. 2:**
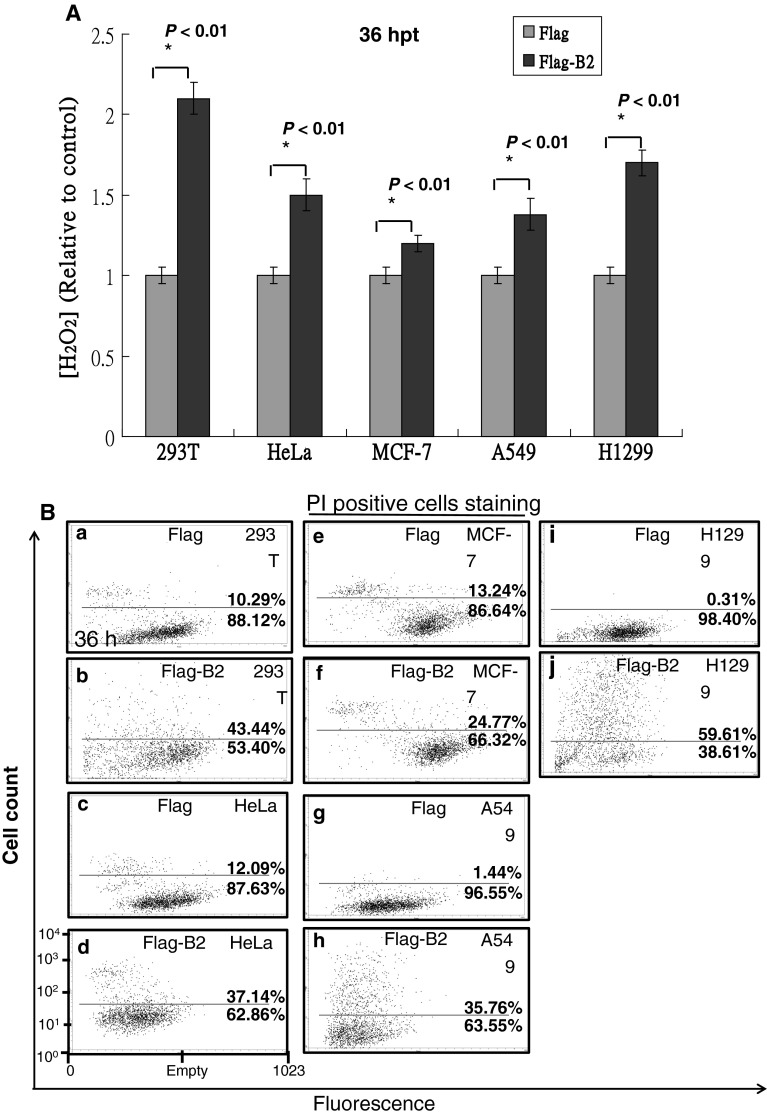
B2 protein induces H_2_O_2_ production and cell death in cancer cell lines. **A** The ratios of H_2_O_2_-producing cells were counted using a fluorescence microplate reader at 36 h. Error bars represent the SEM. All data were analyzed using either a paired or unpaired Student’s *t* test, as appropriate. *P < 0.01. **B** Representative flow cytometric profiles at 36 h post-transfection. PI staining fluorescence was measured from 10,000 cells. Numbers in subpopulation cells (PI^+^) show late apoptotic secondary necrotic cell percentages. The percentage of viable cells (PI^+^) in the major and sub-major population was shown. The Y-axes indicate the cell numbers

To elucidate the mechanism by which B2 protein induces cell death through ROS production, we measured intracellular H_2_O_2_ levels in human 293T cells and in HeLa, MCF-7 A549, and H1299 cancer cell lines (Fig. 2A). At 36 h post-transfection, overexpression of the full-length B2 protein significantly increased intracellular H_2_O_2_ levels in 293T cells (2.1-fold), HeLa cells (1.5-fold), MCF-7 cells (1.2-fold), A549 cells (1.4-fold), and H1299 cells (1.7-fold) compared to cells expressing vector alone (all *P* < 0.01). Cell viability analysis, as determined by PI staining assay, showed that full-length B2 protein can induce cell death in up to 33 % of 293T cells, 25 % of HeLa cells, 11 % of MCF-7 cells, 34 % of A549 cells and 59 % of H1299 cells relative to cells expressing the vector alone at 72 h post-transfection (Fig. 2B), which suggested that cell death was induced via H_2_O_2_ production.

### *N*-acetylcysteine (NAC) suppresses B2-induced cell death in fish cells by inhibiting H_2_O_2_ production

**Fig. 3:**
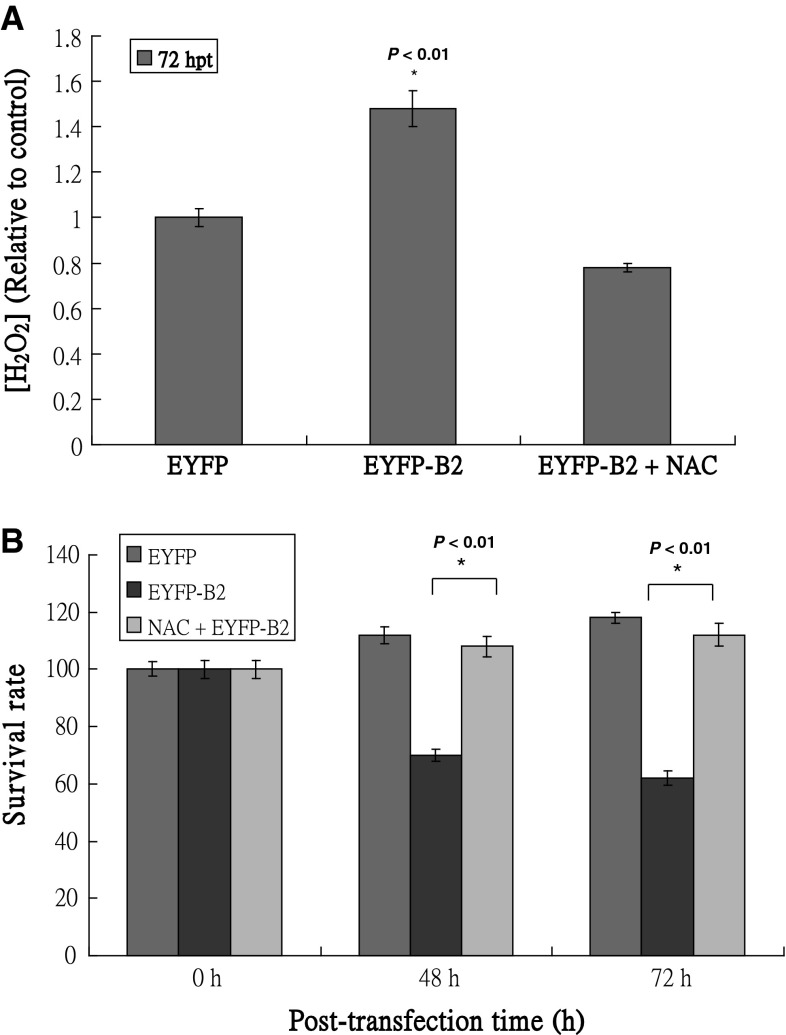
Effect of NAC treatment on H_2_O_2_ production in fish cell expressing B2 protein. **A** Concentration of H_2_O_2_ in the medium of EYFP-, EYFP-B2-, and EYFP-B2 + NAC-transfected cells at 72 h post-transfection. H_2_O_2_ concentration at each time point was determined in triplicate. **B** Survival rate assays in the EYFP-, EYFP-B2-, and EYFP-B2 + NAC-transfected cells at 0, 48, and 72 h post-transfection. Survival rate was determined in triplicate using the trypan blue dye exclusion assay [29]. Each data point (10,000 cells) represents the mean viability of three independent experiments ± SEM. Data were analyzed using either a paired or unpaired Student’s *t* test, as appropriate^.^ *P < 0.01

To determine whether inhibiting H_2_O_2_ production can rescue cells from EYFP-B2-induced death, we assessed cell death in the presence of the anti-oxidant, NAC. As shown in Fig. 3A, EYFP-B2-expressing cells induced up to a 1.5-fold increase in H_2_O_2_ production over cells expressing EYFP alone (*P* < 0.01), which was inhibited in the presence of NAC (0.8-fold) at 72 h post-transfection. Treatment of EYFP-B2-expressing cells with NAC increased cell survival to levels observed in cells expressing EYFP alone at both 48 and 72 h post-transfection (Fig. 3B), which was significantly greater than that observed in EYFP-B2-expressing cells (*P* < 0.01).

### Overexpression of SOD and catalase can reduce B2-induced cell death in fish cells by suppressing H_2_O_2_ production

**Fig. 4:**
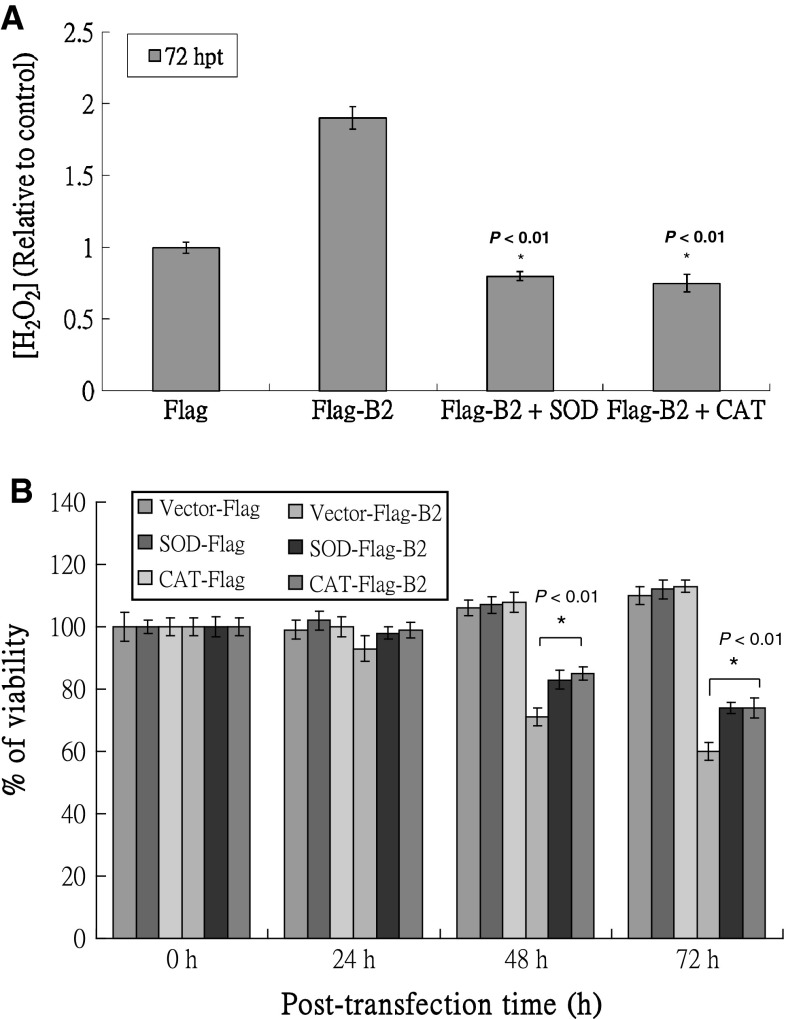
Overexpression of zebrafish Cu/Zn SOD and catalase reduced B2-induced cell death in GF-1 cells by suppressing H_2_O_2_ production. **A** H_2_O_2_ production by GF-1, zebrafish Cu/Zn SOD and catalase-producing cell lines transfected with Flag or Flag-B2 at 72 h post-transfection as determined using a fluorescence microplate reader. *Error bars* represent the SEM. **B** Cell viability of vector control, zfSOD- and zfCatalase-transduced GF-1cells transfected with or without Flag-B2 at 0, 24, 48 and 72 h post-transfection in triplicate using a trypan blue dye exclusion assay. The data were analyzed using either a paired or unpaired Student’s *t* test, as appropriate. *P < 0.01

To determine whether anti-oxidant enzymes can block H_2_O_2_ production and affect host cell viability, zebrafish cells overexpressing Cu/Zn SOD and catalase were selected; the transfection efficiency was approximately 40 %. As shown in Fig 4A, cells overexpressing full-length B2 protein had 1.8-fold increased H_2_O_2_ production; H_2_O_2_ production in cells expressing zfCu/Zn SOD and zfCatalase was 0.8- and 0.7-fold that observed for cells expressing vector alone at 72 h post-transfection, respectively. Furthermore, the viability of cells expressing full-length B2 was increased by approximately 5, 12 and 14 % with zfCu/Zn SOD expression and 6, 14 and 18 % with zfCatalase expression at 24, 48, and 72 h post-transfection, respectively (*P* < 0.01; Fig. 4B). These results are consistent with those shown in Figs. 3 and 4A, demonstrating that H_2_O_2_ stress damage induced by B2 protein could be rescued by either NAC or the antioxidant enzymes, zfCu/Zn SOD and zfCatalase.

### B2 protein expression induces H_2_O_2_ production and up-regulation of anti-oxidant enzymes and Nrf2 via oxidative stress in early zebrafish embryos

**Fig. 5:**
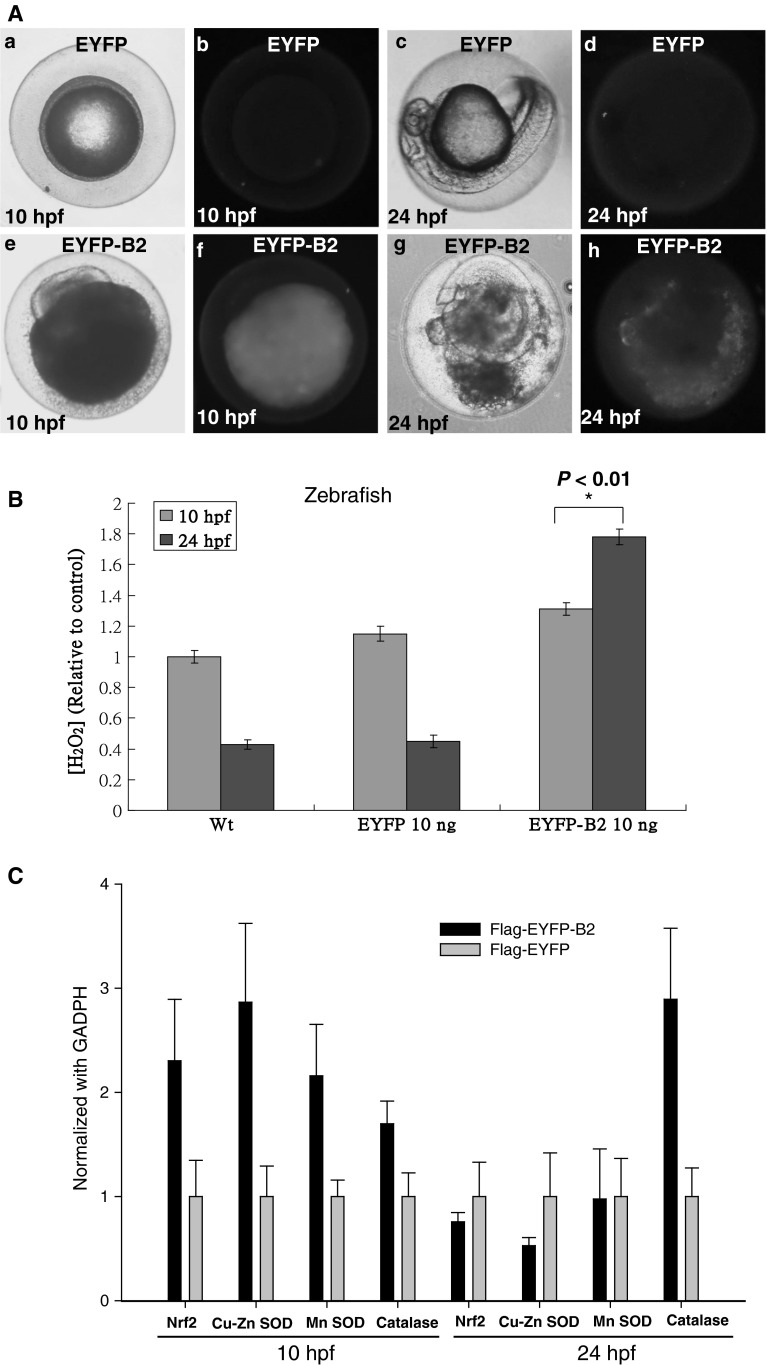
B2 protein induces H_2_O_2_ production, oxidative stress, and zebrafish embryonic death. Embryos were injected with vector control (EYFP) or the *B2* gene (EYFP-B2) at the one-cell stage. **A** Phase-contrast images of the EYFP (a, c) and the EYFP-B2 (10 ng/μL) group (e, g) stained with acridine orange at 10 (b, f) and 24 h post-fertilization (d, h). **B** Intracellular H_2_O_2_ concentrations (fold-increase over vector control) of zebrafish embryos at 10 and 24 h post-fertilization in triplicate. The data were analyzed using either a paired or unpaired Student’s *t* test, as appropriate. *P < 0.01. **C** Up-regulation of Nrf2, Cu-Zn SOD, Mn SOD, and catalase using qRT-PCR after EYFP and EYFP-B2 transfection at 10 and 24 h post-transfection

To establish whether B2 protein is directly responsible for H_2_O_2_-dependent cell death in vivo, the effect of B2 protein expression in zebrafish embryos was examined. Because embryos injected with high doses (30 ng/μL) of pEYFP-B2 did not survive 10 h post-fertilization (hpf), lower doses (10 ng/μL) were employed. The EYFP-B2 fusion protein caused abnormal development at 10 hpf (Fig. 5A(e, f)) that was lethal at 24 hpf (Fig. 5A(g, h)) via cell death. Compared with EYFP-injected embryos (Fig. 5A(b, d)), EYFP-B2-injected embryos showed apoptotic cell accumulation (Fig. 5A(f, h)).

ROS production in injected embryos was next determined. EYFP-B2 fusion protein expression caused rapid increases in intracellular H_2_O_2_ levels of approximately 1.3-fold (at 10 hpf) and 1.78-fold (at 24 hpf) as compared to vector alone (Fig. 5B). Furthermore, qRT-PCR analysis indicated that B2-induced oxidative stress upregulated Nrf2 (2.3-fold), Cu/Zn SOD (2.9-fold), Mn SOD (2.2-fold) and catalase (1.7-fold) expression at 10 hpf (Fig. 5C). However, at 24 hpf, only catalase (1.9-fold) was upregulated as compared to the vector-only control (Fig. 5C). These results suggest that B2 protein expression caused cell death in vivo by increasing H_2_O_2_ production, thus triggering oxidative stress.

### B2 induced-mitochondria fragmentation is correlated with Drp1 recruitment into mitochondria (DRMC)

**Fig. 6:**
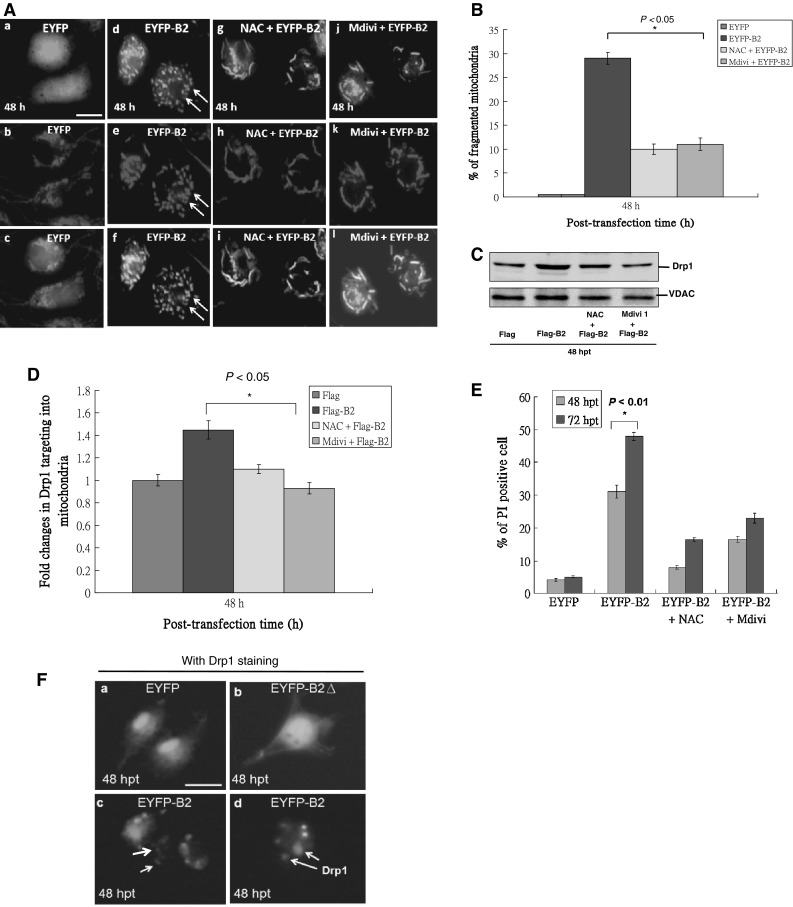
Influence of RGNNV B2-induced H_2_O_2_ production on mitochondrial morphology and cell death in GF-1 cells. GF-1 cells were transfected with EYFP or EYFP-B2 and treated with and without NAC or Mdivi for 48 h and 72 h. **A** Intracellular mitochondrial fragmentation (*scale bar* 10 μm) was determined by fluorescence microscopy, and **B** the induced fragmented mitochondrial ratio was determined in triplicate by individual analysis from **A**. **C** Drp1 translocation to mitochondria was blocked by either NAC or Mdivi treatment as determined by Western blot analysis of mitochondrial fractions. **D** Drp1 mitochondrial targeting from **C** was quantified by densitometry (Molecular Dynamic). **E** Cell viability was determined using the PI assay. **F** Co-localization analysis of protein B2 with Drp1 protein at 48 h post-transfection was detected in approximately 4–5 % cells with weak *blue fluorescence* (**F**(c); indicated by *arrows*) and with stronger blue fluorescence (**F**(d); indicated by *arrows*), which means at different cellular stages compared with EYFP- (**F**(a), staining of Drp1 alone) and EYFP-B2Δ- (**F**(b), with Drp1 staining) transfected cells. All data were analyzed using either paired or unpaired Student’s *t* tests as appropriate. *P < 0.01

Changes in mitochondrial morphology in EYFP- B2-transfected cells were monitored using fluorescence microscopy. B2 protein was localized in the mitochondria and induced mitochondrial fragmentation in cells at 48 h (Fig. 6A(d–f)). Mitochondrial fragmentation was reduced with NAC (Fig. 6A(g–i)) or Mdivi (Fig. 6A(j–l)), indicating that H_2_O_2_ signaling can affect mitochondrial morphology. As shown in Fig. 6B, the proportion of fragmented mitochondria was reduced by 19 and 18 % with NAC and Mdivi treatment, respectively as compared with untreated EYFP-B2-expressing cells (29 %; *P* < 0.05).

Western blot analysis also revealed that localization of full-length B2 to mitochondria results in the recruitment of Drp1 into the mitochondria (Fig. 6C), which was blocked by treatment with NAC (Fig. 6C, lane 3) and Mdivi (Fig. 6C, lane 4) as compared to the untreated group (Fig. 6C, lane 2) and negative control group (Fig. 6C, lane 1). Specifically, Drp1 recruitment to mitochondria was reduced by 0.35- and 0.52-fold with NAC and Mdivi treatment, respectively relative to the Flag-B2 group (*P* < 0.05; Fig. 6D).

Analysis of cell viability with PI staining indicated that cell death was decreased by 23 and 14 % with NAC and Mdivi treatments, respectively compared to those without inhibitors at 48 h (Fig. 6E). After 72 h, cell death was further decreased by 31 and 24 % with NAC and Mdivi treatments, respectively (Fig. 6E), which correlated to Drp1 recruitment to mitochondria.

To determine if B2 protein and Drp1 co-localized in mitochondria, immunofluorescence analysis was undertaken. At 48 h post-transfection, approximately 4–5 % of the cells were strongly damaged (Fig. 6F(c, d); indicated by arrows). In these damaged cells, EYFP-B2 (green) and Drp1 (blue) were co-localized (6F: d) when compared with Drp1 staining in either EYFP- or EYFP-B2Δ-expressing cells (Fig. 6F(a, b)), which did not shown any blue image as a normal controls.

## Discussion

The present study demonstrates that the viral B2 protein induces H_2_O_2_-mediated cell death through pathways that may involve the host anti-oxidant enzyme system and Drp1-mediated mitochondrial fragmentation. The RGNNV B2 protein induced H_2_O_2_ production in fish cells, human cancer cells, and embryonic zebrafish in a similar fashion in vitro and in vivo. The p53-null lung cancer cells (H1299) were more severely damaged than those expressing p53 (A549), resulting in a 0.3-fold increase in H_2_O_2_ production and a 25 % increase in cell death. Furthermore, NAC dramatically reduced H_2_O_2_ production by 0.7-fold, and overexpression of zfCu/Zn SOD and zfcatalase decreased H_2_O_2_ levels by 0.6- and 1.0-fold, respectively, which coincided with increased cell viability at 72 h post-transfection. These results indicate that antioxidants and antioxidant enzymes may have therapeutic potential for preventing B2-protein-induced cell damaged.

B2-induced oxidative stress can up-regulate the antioxidative enzymes, Mn SOD, catalase, and Nrf2, and RGNNV-induced H_2_O_2_ signaling may modulate viral replication in the early and middle stages (24–48 h post-infection). Thus, we propose that the H_2_O_2_ balance is severely disrupted during late replication by viral death inducers [6, 8, 22] that cause H_2_O_2_ production. During the early and middle replication stages, H_2_O_2_-mediated responses may play a dual role, either enhancing viral replication or modulating the oxidative stress response by up-regulating antioxidant enzymes, such as catalase and Cu/Zn SOD [23]. However, the final outcome is dependent on viral death factor expression, such as B2 protein and protein α (capsid protein). In our observation, the advantage of NAC treatment or Cu/Zn SOD and catalase expression in blocking-B2 function is greater than the effects on whole viral infection.

H_2_O_2_ production in early embryos expressing B2 protein increased about 0.3-fold at 10 hpf and 1.3-fold at 24 hpf, which correlated with the increase in Nrf2, Cu/Zn SOD, Mn SOD and catalase expression at 10 hpf, but only catalase at 24 hpf, suggesting that these proteins are quickly up-regulated to metabolize ROS. Nrf2 is a cellular sensor of chemical- and radiation-induced oxidative and electrophilic stress [30] and controls the expression and coordinated induction of a battery of defensive genes encoding detoxifying enzymes and antioxidant proteins. However, it is not known whether Nrf2 up-regulated the anti-oxidant enzymes in our system. Although the up-regulation of these genes may help to restore ROS (H_2_O_2_) homeostasis, it did not protect the embryo from damage induced by B2 overexpression at 10 or 24 hpf.

In the present study, B2-induced mitochondrial fragmentation correlated with Drp1 translocation. Mitochondria form a dynamic cellular network that is tailored to the energetic and metabolic requirements of the cell [31, 32]. The morphology of the mitochondrial network within cells represents a delicate balance between fusion and fission events. Proteins involved in both the regulation and maintenance of mitochondrial morphology have crucial roles in maintaining the health of the cell [31, 33]. The master regulator of mitochondrial fission is a largely cytosolic member of the dynamin family of GTPases, Drp1 in mammals and Dnm1 in yeast [34–37]. Drp1 polymerizes into spirals around mitochondria, and through GTP hydrolysis, it constricts the mitochondrion, leading to membrane scission [38–41]. Drp1 activity at the mitochondrial outer membrane is regulated by a variety of post-translational modifications [42–47] and by interactions with mitochondrial accessory and effector proteins. Changes in Drp1 activity upon phosphorylation may depend on external parameters, such as cell type, age or status, or on internal parameters, such as the localization of Drp1. These kinases phosphorylate and dephosphorylate multiple protein targets, further complicating the interpretation of the role of Drp1 [47].

In our study, B2 protein overexpression induced mitochondrial fragmentation in a homogeneous manner. Such damage was prevented by NAC and the Drp1 inhibitor, Mdivi, which correlated with a reduction in the fragmented mitochondria ratio (Fig. 6B). We also observed increased Drp1 translocation to the mitochondria upon B2 protein expression, which was blocked by NAC and Mdivi. In addition, co-localization of B2 protein and Drp1 was observed at 48 h post-transfection, indicating that Drp1 translocation is triggered by H_2_O_2_ signaling. Whether phosphorylation or other modifications of Drp 1 occur during this process is still unknown.

**Fig. 7:**
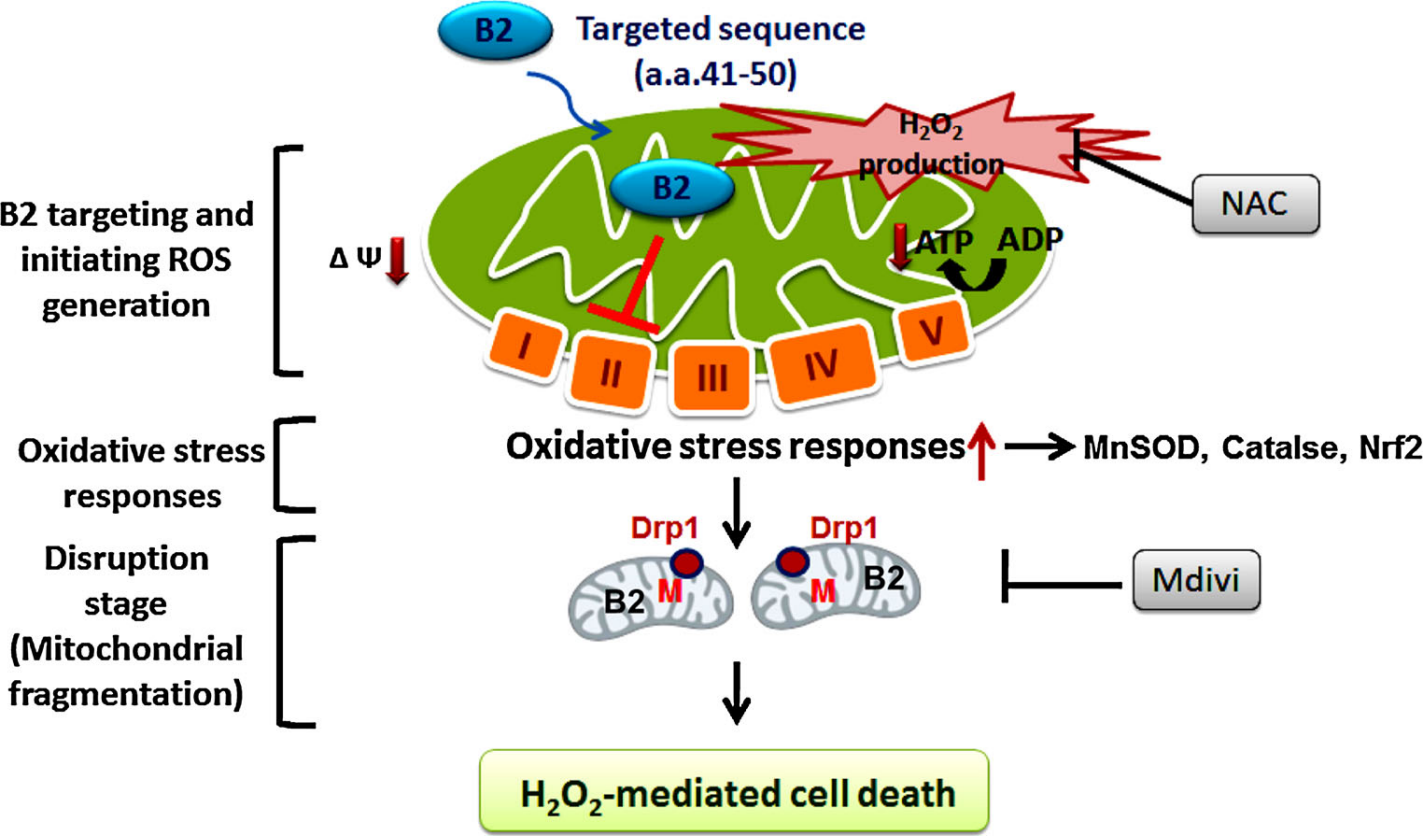
Schematic illustration of a proposed mechanism by which protein B2 induces H_2_O_2_-mediated cell death in B2-transfected cells. In RGNNV B2-transfected cells, protein B2 induces Drp1-mediated mitochondrial fragmentation and cell death. B2 protein is targeted to the mitochondria via its amino acid targeting signal, ^41^RTFVISAHAA^50^ [27] where it modulates complex II activity, inducing H_2_O_2_ production. In response to the resulting oxidative stress, the expression of antioxidant enzymes that can metabolize the accumulated H_2_O_2_ is upregulated. Finally, cells enter an unbalanced state of higher H_2_O_2_ production, inducing the translocation of the mitochondrial fission factor, Drp1, to the mitochondria, triggering mitochondrial fragmentation and cell death

In summary, the betanodavirus B2 protein is translocated to the mitochondria via a specific targeting sequencing in its N-terminus (Fig. 7). Once in the mitochondrial matrix, the B2 protein can block complex II function, resulting in loss of mROS homeostasis and depletion of ATP [48]. The increase in H_2_O_2_ causes oxidative stress, leading to the upregulation of antioxidant enzymes and transcription factors, such as Mn SOD, catalase, and Nrf2. Finally, H_2_O_2_ signals can induce the recruitment of the mitochondrial fission protein, Drp1, into the mitochondria, where it participates in mitochondrial fragmentation and triggers oxidative stress-mediated cell death.
